# MIH and Dental Caries in Children: A Systematic Review and Meta-Analysis

**DOI:** 10.3390/healthcare11121795

**Published:** 2023-06-18

**Authors:** Marta Mazur, Denise Corridore, Artnora Ndokaj, Roman Ardan, Iole Vozza, Sylvie Babajko, Katia Jedeon

**Affiliations:** 1 Department of Oral and Maxillofacial Sciences, Sapienza University of Rome, 00161 Rome, Italy; marta.mazur@uniroma1.it (M.M.); denise.corridore@uniroma1.it (D.C.); iole.vozza@uniroma1.it (I.V.); 2 Department of Economic Sciences, Koszalin University of Technology, 75-343 Koszalin, Poland; roman.ardan@tu.koszalin.pl; 3 Laboratory of Biomedical Research in Odontology, Unité Propre de Recherche 2496, Université Paris Cité, 1 rue Maurice Arnoux, 92120 Montrouge, France; sylvie.babajko@inserm.fr (S.B.); katia.jedeon@u-paris.fr (K.J.); 4 Department of Restorative Dentistry and Endodontics, Rothschild Hospital, 5 rue Santerre, 75012 Paris, France

**Keywords:** MIH, molar incisor hypomineralization, dmft, DMFT, caries, caries experience, biostatistics, dental public health, epidemiology

## Abstract

(1) Background: Molar-incisor hypomineralization (MIH) is a clinical condition affecting permanent teeth in children, with a documented rising trend in the last two decades. The aim of the present study was to analyze and synthesize the available evidence on caries experience (dmft/DMFT) and MIH in children. (2) Methods: A systematic review and meta-analysis were conducted according to the PRISMA statement. (3) Results: 59 papers published between 2007 and 2022 were included in the qualitative synthesis and 18 in the meta-analysis. The total sample of subjects was 17,717 (mean: 896), of which 2378 (13.4%) had MIH (mean: 119), with a girl/boy ratio of 1:1. The mean age of the enrolled participants was 8.6 (age range 7–10 years). Meta-analysis showed that MIH has a positive correlation with both dmft (effect size of 0.67, 95% CI [0.15, 1.19]) and DMFT (effect size of 0.56, 95% CI [0.41, 0.72]); (4) Conclusions: Children with MIH should be diagnosed correctly and on time. Treatment and management options for moderate and severe forms of MIH should consider prognosis based on known risk factors, and secondary and tertiary prevention policies should also consider the multifactorial nature of caries etiology.

## 1. Introduction

In 2001, Weerheijim and members of the European Academy of Pediatric Dentistry (EAPD) coined the term “Molar Incisor Hypomineralization” (MIH) to define an enamel defect of systemic origin that affects one to four first permanent molars and can possibly affect permanent incisors. The first study in which the clinical features were described was published in 2003 [1].

Elfrink et al. [2] in 2008 proposed the use of the term “Deciduous Molar Hypomineralization” (DMH) if a hypomineralization defect was found on at least one deciduous second molar; furthermore, the same author stated that the presence of the defect in the deciduous dentition greatly increases the likelihood of MIH in the permanent dentition [2].

Although MIH refers to defects in permanent first molars and incisors, coexisting lesions have also been observed in the cusps of canines and permanent second molars and premolars. The term MH (Molar Hypomineralization) is used when there is no incisor involvement [3,4].

The etiology described is multifactorial, with systemic, genetic, and epigenetic factors acting synergistically or additively [5], and it is attributable to maternal and child health in perinatal life. In this scenario, particular attention should be paid to the development of the oral microbiome in early life [6] and the concomitant presence of syndromes and genetic mutations affecting the orofacial structures [7].

Clinically, the enamel of the affected teeth presents qualitative or quantitative defects, or a combination thereof. The colorimetric alterations can vary from creamy-white defects with diffuse margins to yellow and brown defects with demarcated margins [8]. These observed alterations have been defined as a prognostic factor for the altered teeth; in fact, when yellow and brown defects are present within the first permanent molars, Cabral et al. predicted post-eruptive breakdown (PEB) within one year after eruption (i.e., the collapse of the occlusal chewing surface with exposure of dentin and increased clinical signs such as pain and hypersensitivity with increased treatment need [3,9,10]. The worldwide prevalence of MIH varies, according to the literature, between different geographical macro-areas [5].

Data on prevalence variability may be explained by the different methods and/or indexes of diagnosis used and the inhomogeneous ages and populations present in the epidemiological studies. Furthermore, in those populations with a high caries prevalence, this may mask the actual prevalence of MIH [11,12,13].

In 2018, a systematic review by Schwendicke et al. determined that the global burden of MIH is highly prevalent worldwide, with an estimated average prevalence of 13.1%, affecting approximately 878 million people. There are 17.5 million new cases each year, of which 27.4% require treatment for pain, hypersensitivity, or PEB [10].

A moderately or severely damaged first permanent molar (FPM) or an aesthetically compromised upper central permanent incisor that nevertheless has a chance of being retained in the oral cavity in the medium or long term requires rapid and effective intervention through direct or indirect minimally invasive techniques (resin infiltration, sealants); also, topical application of fluoride gels and sugar substitutions appear to be useful for reducing dental caries [14,15,16,17,18].

The choice of material and technique must consider the severity and extent of the defect, the quality of the affected and sound enamel, the presence of hypersensitivity, the patient’s age, and his or her level of cooperation.

Children with MIH have been shown to undergo four times more dental treatment during adolescence than control groups. Furthermore, children with MIH present behavioral problems compared to the control group, which may be associated with the hypersensitivity of the dental elements affected by MIH and the pain and anxiety of treatment, as these elements often cannot be adequately anesthetized [9]. The presence of severe forms of MIH in the FPMs has a high impact on the quality of life of the patient and the family unit. The economic burden of treatment must also be considered, as the prevalence of MIH is characterized by an increasing trend, with the most severe forms remaining untreated in most cases [12]. The decision to treat a molar with severe MIH early on has long-term consequences both clinically and economically. Some treatments, such as sealants and composite resins, are initially much less costly than others, such as indirect restorations or orthodontic removal and alignment. However, they may require more restorations and earlier tooth loss, resulting in higher long-term costs [19].

The aim of this manuscript was to systematically review and synthesize available epidemiological observational studies investigating the prevalence of caries among children with and without MIH. The results of the present review are aimed at providing useful information to make accurate diagnostic and clinical decisions for children with MIH.

## 2. Materials and Methods

The PRISMA (Preferred Reporting Items for Systematic Reviews and Meta-Analyses) statement [20] and the Cochrane Handbook for Systematic Reviews of Interventions [21] guidelines were used for this systematic review. A PICO (Population, Intervention, Comparison, and Outcome) question was utilized to formulate a focused question and guide the inclusion and exclusion criteria of the present study. The registration on PROSPERO was performed after the screening stage (CRD42022375575).

### 2.1. Eligibility Criteria

The following inclusion criteria were applied for this search: (a) in-vivo studies; (b) peer-reviewed observational studies, regardless of the type (cross-sectional, cohort, case-control); (c) all considered participants were children; (d) studies reporting MIH (i.e., a described defect of permanent molars with or without affected permanent incisors); (e) MIH had to be defined according to the European Academy of Paediatric Dentistry (EAPD) definition or its modifications [22,23]; (f) studies published in English, Italian, French, Spanish, Albanian, and Polish.

The exclusion criteria were (a) in-vitro studies; (b) absence of effective statistical analysis, where an attempt was made to ask the authors about it, without receiving any response; (c) debates, editorials, and abstracts; (d) studies that assessed the prevalence of enamel defects not restricted to MIH (e.g., diffuse opacities) and that did not allow extraction of the MIH component; (e) studies reporting only primary molars; (f) systematic reviews and meta-analyses were excluded.

The PICO question was as follows: (1) Population: children; (2) Intervention: presence of MIH; (3) Comparison: absence of MIH; and (4) Outcome: caries experience in participants with or without MIH.

### 2.2. Search Strategy and Study Selection

The literature search for free-text and MeSH terms was conducted on PubMed, Google Scholar, and Scopus from 2002 to 30 July 2022. A combination of subject headings and free-text terms was used. Finally, the search strategy was determined by several preliminary searches. The keywords used in the search strategy were as follows: (“molar incisor hypomineralization” [All Fields] OR “MIH” [All Fields]) AND “prevalence” [All Fields] AND (“children” [All Fields] OR “schoolchildren” [All Fields]) AND (“caries experience” [All Fields] OR (“DMFT” [All Fields] AND “dmft” [All Fields])).

Reference lists of primary study reports were cross-checked to find further studies. Guided by the inclusion criteria, two authors (MM and AN) independently screened the literature by reading the titles and abstracts. The full text of each identified article was then read to determine its suitability for inclusion. Disagreements were resolved by consensus or by discussion with a third author (KJ).

### 2.3. Data Collection

For each study, data were extracted independently by two authors (MM and AN) and analyzed by a third author (KJ) by creating a piloted spreadsheet and comparing them through it, according to Cochrane Collaboration guidelines [13]. For missing data, MM contacted the author of the research by email and excluded those for which there was no response.

### 2.4. Data Items

The collected data items were: year of screening, country, city, continent, setting (rural/urban), screening setting (school/university/hospital), total sample, sample with MIH, total girls/girls with MIH, total boys/boys with MIH, age range and mean age in all screened groups (whole sample, MIH sample, control sample, boys’ and girls’ groups), MIH definition, MIH severity, total number of teeth affected, molars/incisor ratio, dmft, and DMFT (whole sample, MIH sample, control sample, boys’ and girls’ groups).

### 2.5. Quality Assessment

As indicated by PRISMA, a methodological quality assessment provides an indication of the strength of the evidence provided by a study, as methodological flaws can lead to bias. For cross-sectional studies, according to the Newcastle-Ottawa Scale (NOS), the quality assessment score can range from zero to nine points, with a high score indicating a good-quality study [24].

### 2.6. Risk of Bias in Individual Studies

Selection bias (consecutive or random sample of patients enrolled), performance and detection bias (participant and operator blinding), attrition bias (patient drop-out, missing values or participants, and follow-up time that is too short), and reporting bias (selective reporting, unclear elimination, missing results) were identified, assessed, and attributed according to Cochrane guidelines [21].

### 2.7. Summary Measures and Heterogeneity

Heterogeneity was assessed quantitatively using I^2^-statistics and Cochran’s Q [25]. The results were considered statistically significant at *p* < 0.05. Publication bias was estimated using a funnel plot and applying Egger’s test of its asymmetry [26].

### 2.8. Statistical Analysis

Meta-analysis was performed with the R statistical program, v. 4.2.1 (The R Foundation for Statistical Computing, Wirtschaftsuniversität Wien, Vienna, Austria), using a random-effect model via the metafor R package [27]. The mean difference (MD) for DMFT and dmft level were calculated as effect estimates.

The influence of individual studies was verified using the difference in fit (DFFITS) diagnostic, Cook’s distance, and leave-one-out meta-analysis to assess how each individual study affected the overall estimate. 95% confidence intervals were used to demonstrate levels of uncertainty.

## 3. Results

### 3.1. Study Selection

**Figure 1:**
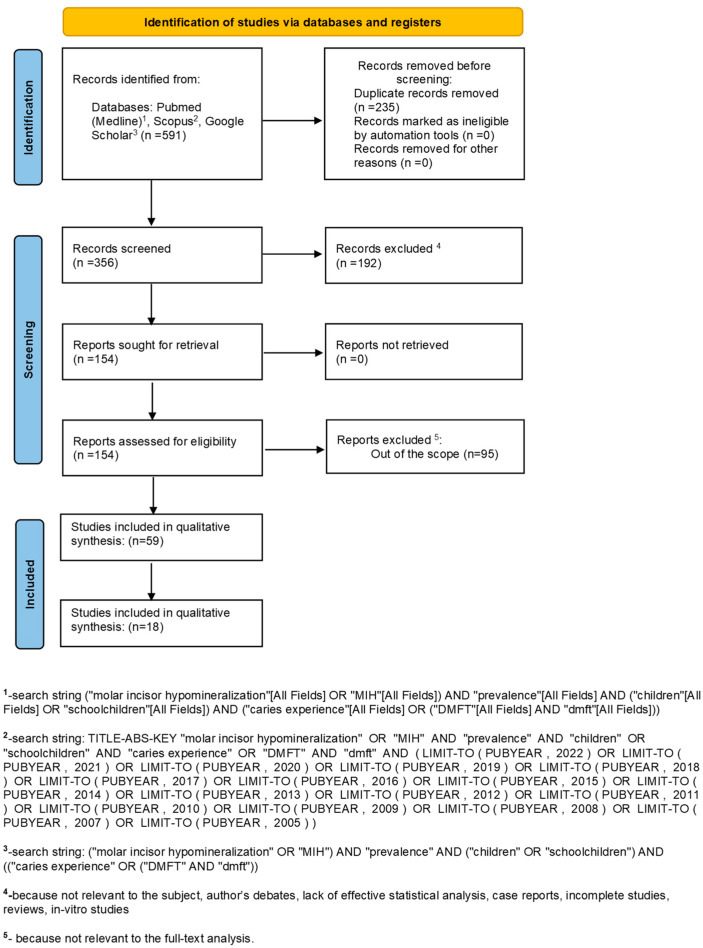
The flow diagram of the search.

The search strategy identified 591 potential articles: 148 in PubMed, 74 in Scopus, and 369 in Google Scholar. After eliminating duplicates, 356 articles were first screened. Subsequently, 192 articles were excluded because they did not meet the inclusion criteria. Of the remaining 154 articles, 95 were excluded because no relevance to the topic of the study was found. The remaining 59 articles were included in the qualitative synthesis and 18 in the meta-analysis (Figure 1). Table S1 summarizes the characteristics of each of the 18 included studies.

### 3.2. Study Characteristics

The included studies (Table S1) [12,28,29,30,31,32,33,34,35,36,37,38,39,40,41,42,43,44] were published between 2007 and 2022 and were all cross-sectional studies. The total sample size was 17,717 participants (mean: 896), of which 2378 (13.4%) had MIH (mean: 119). Not all studies had the description number of girls and boys, so the total number of girls described by most studies was 8861 (mean: 466), of which 1062 (mean: 59) had MIH, while the total number of boys was 8203 (mean: 432), of which 972 (mean: 54) had MIH. The mean age of the enrolled participants was 8.6 (mean age range 7–10). The reported mean dmft index was 3.80 ± 3.22, where the mean dmft for participants with MIH was 4.25 ± 2.63 compared to 3.59 ± 2.70 for controls. The reported mean DMFT was 1.16 ± 1.27, where the mean DMFT for participants with MIH was 1.50 ± 1.14 and the mean DMFT for controls was 0.92 ± 1.00.

### 3.3. Quality Assessment

According to the Newcastle-Ottawa scale (NOS) for cross-sectional studies (n: 59) [12,28,29,30,31,32,33,34,35,36,37,38,39,40,41,42,43,44,45,46,47,48,49,50,51,52,53,54,55,56,57,58,59,60,61,62,63,64,65,66,67,68,69,70,71,72,73,74,75,76,77,78,79,80,81,82,83,84,85], the authors evaluated the qualities of all included studies based on object selection, comparability, and exposure. A star was described as an appropriate entry, with each star representing one point. The possible quality assessment score ranged from zero to nine points, with a high score indicating a good-quality study. In the evaluation of the quality of cross-sectional studies, the total score in all 59 cases was greater than or equal to 6, indicating high-quality studies (Table S2).

### 3.4. Risk of Bias Assessment

The majority of studies included in the quantitative synthesis (88%) used random or comprehensive sampling [12,28,29,30,31,32,34,35,36,40,41,42,43,44]; only one study (5%) stated that the participants were blinded, indicating low risk for performance and detection bias [30], while all the other studies (95%) presented high risk of bias [12,28,29,31,32,33,34,35,36,37,38,39,40,41,42,43,44]; four studies (22%) presented high risk for attrition bias [29,33,40,42]; finally, all the included studies presented low risk for reporting bias [12,28,29,30,31,32,33,34,35,36,37,38,39,40,41,42,43,44] (Table S3).

### 3.5. Meta-Analysis

18 studies reported data on the Decayed, Missing, and Filled Teeth (DMFT) index [12,28,29,30,31,32,33,34,35,36,37,38,39,40,41,42,43,44] and 12 for dmft [12,32,33,34,35,36,37,38,40,41,43,44], with data sufficient for meta-analysis to assess differences in caries experience among patients with and without MIH.

#### 3.5.1. MIH and DMFT

**Figure 2:**
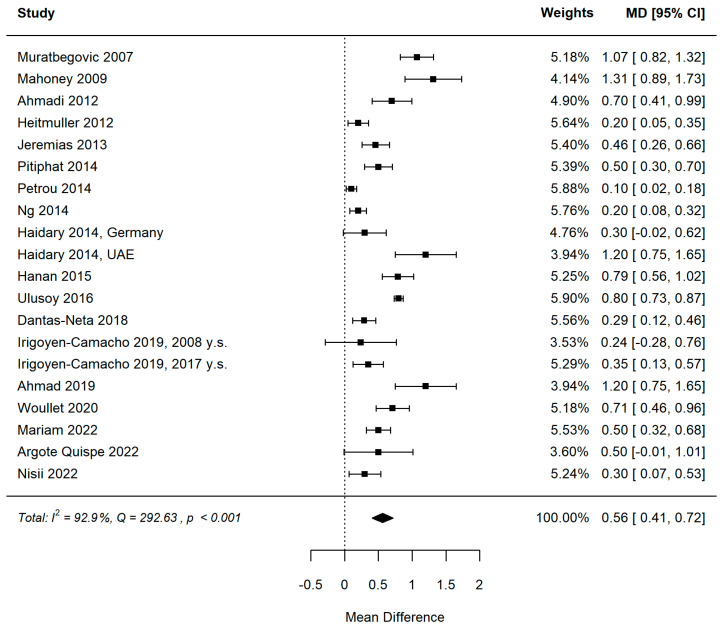
Forest plot for DMFT. A positive value of the mean difference means a higher DMFT level in the MIH group [12,28,29,30,31,32,33,34,35,36,37,38,39,40,41,42,43,44].

**Figure 3:**
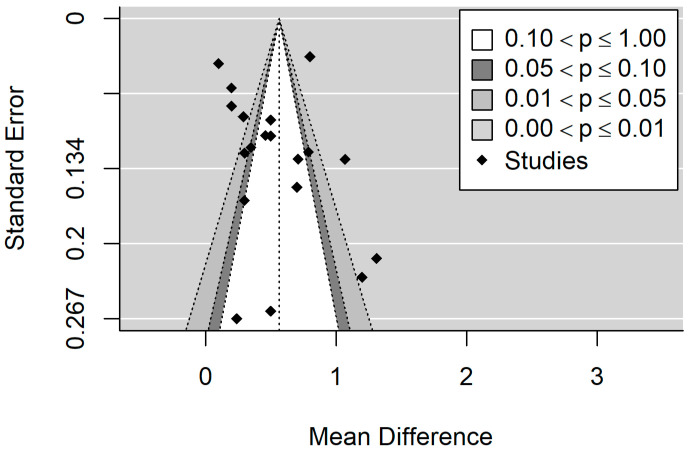
Funnel plot for DMFT. The symmetrical pattern in the funnel plot is indicative of publication bias absence.

Figure 2 and Figure 3 show the forest plot and funnel plot for DMFT. The MIH disease has a significant (*p* < 0.001) positive effect on DMFT level (higher mean DMFT level in the MIH group). Study results are inconsistent; heterogeneity is significant (*p* < 0.001), with about 93% of the variability coming from heterogeneity. Egger’s test indicates funnel plot asymmetry (*p* = 0.026), which can be caused by publication bias.

**Figure 4:**
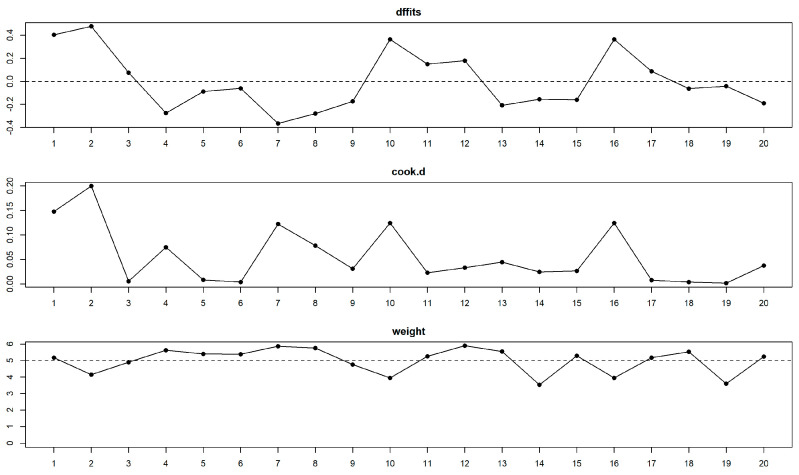
Influence diagnostics did not reveal any influential studies for the DMFT meta-analysis. “dffits” is for difference in fit diagnostic, “cook.d” for Cook’s distance, and “weight” plot corresponds to study weights in the meta-analysis.

Influence analysis did not reveal any influential studies in the DMFT meta-analysis (Figure 4). Leave-one-out meta-analysis confirmed the yielded pooled estimates to be robust, with partial leave-one-out estimates in the [0.528–0.590] range and significant (all partial *p* < 0.001).

#### 3.5.2. MIH and dmft

**Figure 5:**
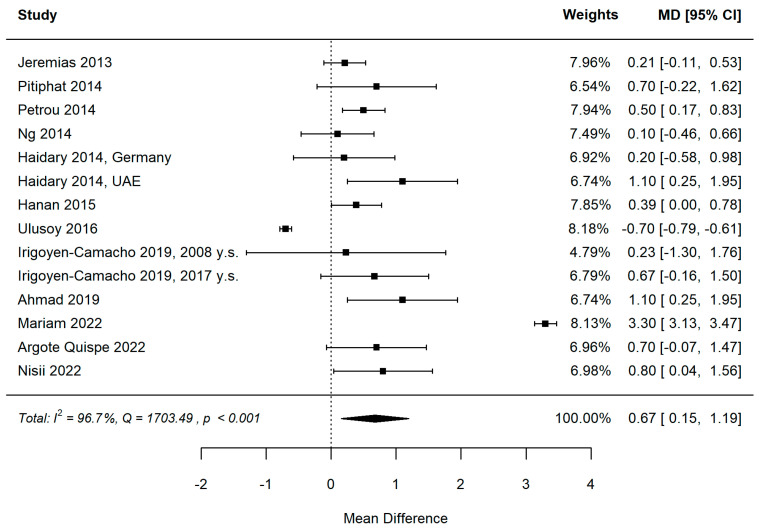
Forest plot for dmft. A positive value of the mean difference means a higher mean dmft level in the MIH group [12,32,33,34,35,36,37,38,40,41,43,44].

**Figure 6:**
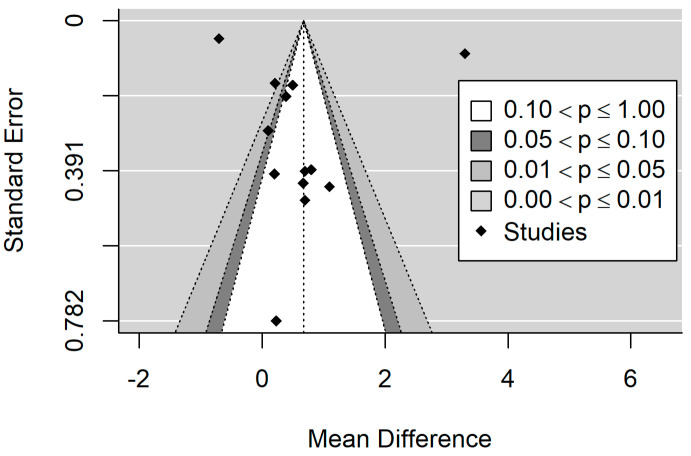
Funnel plot for dmft. There is no obvious asymmetry in the plot.

Figure 5 and Figure 6 show the forest plot and funnel plots for dmft. The MIH disease has a highly significant (*p* = 0.011) positive effect on dmft level. Study results are inconsistent—heterogeneity is significant (*p* < 0.001), and about 98% of the variability comes from heterogeneity. Egger’s test does not indicate funnel plot asymmetry (*p* = 0.798).

**Figure 7:**
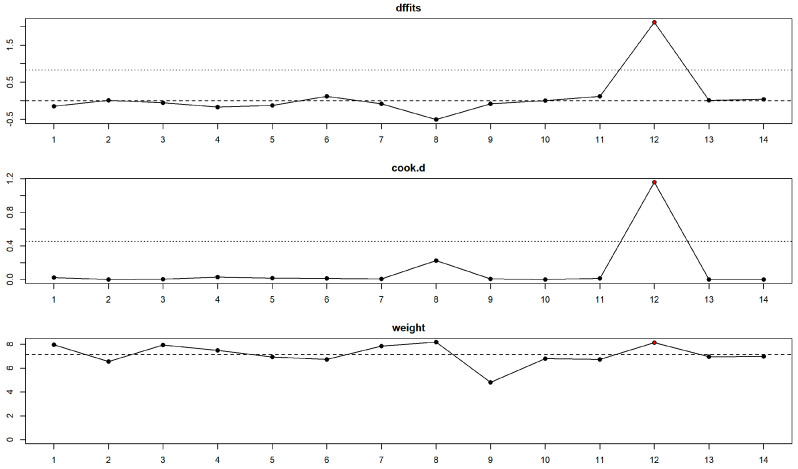
Influence diagnostics revealed one influential study (red point) for the dmft meta-analysis. “dffits” is for difference in fit diagnostic, “cook.d” for Cook’s distance, and “weight” plot presents study weights in the meta-analysis.

Influence analysis reveals one influential study [43] in the dmft meta-analysis (Figure 7). The study reported a very high positive effect size. Leave-one-out meta-analysis resulted in a lower but still significant pooled effect size of 0.389 (*p* = 0.015) in the case of study out [43] and confirmed the yielded pooled estimates to be robust with partial leave-one-out estimates in the [0.643–0.800] range and significant (all partial *p* < 0.022) in other cases.

## 4. Discussion

This systematic review aimed to highlight the experience of caries in children with and without MIH. Fifty-nine articles were included in the qualitative evaluation and 18 in the meta-analysis. It was shown that MIH has a positive correlation with both dmft (effect size of 0.67, 95% CI [0.15, 1.19]) and DMFT (effect size of 0.56, 95% CI [0.41, 0.72]), from a total of 17,717 subjects.

The results of this systematic review are not uniformly reflected in the literature, as there are studies both confirming and denying the presence of a correlation between MIH and dental caries.

For example, the study by Lago et al. [86] showed no significant correlation between MIH and primary or permanent dentition in a group of 545 schoolchildren aged 6–12 over a period of 6 years. By contrast, the results of Salem et al. [87] indicated a positive correlation between children with MIH and DMH and caries in permanent dentition, but the authors also suggested caution when interpreting the results, as they were based on only two clinical studies. These contradictory findings deserve a possible explanation. The diagnosis of MIH picks up enamel defects of varying severity, which may or may not be related to dental hypersensitivity that can act as a block to proper dental hygiene in children. This is why, when there are discrepant results between studies, the severity of the forms of MIH that have been diagnosed should be verified, as in the case of Lago et al., where 82% of the sample consisted of mild forms of MIH [86]. This result could therefore suggest that it is not the mild form of MIH that correlates with dental caries, but the moderate and severe forms.

Molar incisor hypomineralization is a clinical condition that is often underestimated and left untreated. It affects school-age children from the time of eruption of the first permanent molars, although similar forms have been described that can also affect deciduous molars [83,88].

Despite the Global Oral Health Goals for 2020 set by the World Dental Federation, WHO, and the International Association for Dental Research to minimize the impact of dental caries on individuals and society and to formulate efficient strategies for caries prevention, detection, and management [89], caries experience remains prevalent in most countries worldwide, with a dmft ranging from 0.9 to 7.5 among preschool children across the globe [90] and data for 12-year-olds in Europe showing a DMFT ranging from 0.5 in Germany and the United Kingdom to 6.9 in the Republic of North Macedonia [91]. Data show that DMFT scores have been reduced since 2000 in almost all EU member states and that higher GDP (PPS) (gross domestic product in purchasing power standards) is associated with lower DMFT values [92]. The effort to implement strategies to achieve caries-free pediatric populations at a European level will have to consider the increasing trend of MIH prevalence over the last 10 years [12]. For example, Italian data on MIH in 2005 reported a prevalence of 13.7% with all mild forms [93], and in 2022, 18.2% with severe forms in 82.5% of the lesions [12]. This trend was also confirmed by data from extra-European surveys, as in the study by Irigoyen-Camacho et al. that compared the outcomes from 2008 and 2017 cross-sectional studies in Mexico City [40]. In less than ten years, the prevalence of MIH increased from 20.3% to 31.9%, with severe cases more than doubling from 4.3% to 9.8%. The diagnostic detection was performed in both cases by the same operators, thus eliminating the bias due to inter-individual variability, but it must also be considered that the same operators over a period of almost ten years may have increased their clinical skills and experience [40].

The presence of MIH in the oral cavity is correlated with developmental defects in enamel, which can affect one or more first permanent molars and/or permanent incisors. Clinical signs are characterized by qualitative defects in the enamel. In severe cases, post-eruptive breakdown can occur on the occlusal surface of affected teeth, with dentin exposure and subsequent pulpal inflammation. Children presenting with severe occlusal forms can manifest behavioral problems because of the pain and hypersensitivity experienced [94]. Strategies for the clinical management of the more severe forms involve a different clinical approach for the upper arch, where extraction of the affected permanent first molar and favoring orthodontic mesialization of the second molar can be expected between 11 and 13 years of age; and for the lower arch, where more conservative approaches are preferred, through root canal treatment and prosthetic restoration of the MIH-affected teeth [95].

It is probable that the correlation between MIH and increased experience of caries in children is due to the fact that hypersensitivity acts as a barrier to the proper and regular performance of oral hygiene measures at home [96]. A recent clinical study on oral hygiene and gingival health among children with MIH showed that plaque index, gingival index, and bleeding on probing were significantly higher in the MIH group than in the control group, and they also correlated with MIH severity [97]. In fact, Raposo et al. demonstrated in a large sample of children with MIH that dental hypersensitivity affects more than half of the molars with either moderate or severe forms of MIH. The authors also reported that 90% of the molars with MIH and post-eruptive breakdown were affected by caries [98].

Children with moderate and severe forms of MIH should be examined not only for caries risk but also for periodontal defects. In fact, the alteration of these parameters in childhood is prognostic for the adult individual’s oral health [17]. Moreover, the global economic impact of MIH should be assessed, as it is estimated that 27.4% (23.5–31.7%) of all MIH cases (approximately 5 million new cases each year and 240 million existing cases) will require dental treatment due to hypersensitivity and post-eruptive enamel breakdown [10].

Surprisingly, no studies in the literature on dietary counseling in children with MIH were available, even though the clinical picture of the developing patient presenting with moderate and severe forms of MIH characterized by hypersensitivity should also be framed from the point of view of solid foods and beverages in the daily diet to decrease the risk of caries and periodontal inflammation as much as possible.

The results must be carefully interpreted, as selection bias may affect the main outcomes (dmft/DMFT in subjects with/without MIH). Selection bias may occur when the age range of enrolled samples is different, as higher DMFT may be correlated with older populations; when MIH is assessed by different diagnostic criteria; or when the enrolled sample is from a dental clinic or is population-based, as those from dental clinics will be in greater need of treatment. In the present study, two studies (11%) [31,38] were of enrolled children in a dental clinic or general hospital setting, and 16 studies (89%) [12,25,26,27,29,30,31,32,33,34,36,37,38,39,40,41] were in a population-based setting such as schools. Six studies were conducted in Europe [12,28,31,34,38,42], five studies in Asia [30,33,35,41,43], one study both in Europe and Asia [36], one study in Oceania [29], four studies in South America [31,37,39,44], and one study in North America [40]. The presence or absence and gravity of MIH were evaluated in 14 cases (78%) [12,31,32,33,34,35,36,37,38,39,40,41,43,45] by EAPD criteria, with a reported mean DMFT for participants with MIH that was 1.38 ± 1.10, while the mean DMFT for controls was 0.88 ± 0.95, and in four cases (22%) [28,29,30,42] by other criteria, with a reported mean DMFT for participants with MIH that was 2.00 ± 1.28, while the mean DMFT for controls was 1.06 ± 1.24. The age range of enrolled subjects was 6–12; four studies (22%) [12,33,35,40] evaluated 6–8-year-old schoolchildren, while 14 studies (78%) [28,29,30,31,32,34,36,37,38,39,41,42,43,44] examined young adolescents.

The results of the present systematic review and meta-analysis are in accordance with previous works. A systematic review published in 2017 evaluating the correlation between MIH and dental caries over a period from 2003 to 2015 in 14 countries showed a significant association between dental caries and MIH in 17 studies. The sample was smaller than the present one, and the authors warned that caution was needed in interpreting the results. Moreover, the OR was not comparable with the present results, as there was no meta-analysis [99].

A case-control study on children aged 7–13 years presenting with MIH by Grossi et al. showed a higher caries experience in the permanent but not in the deciduous dentition [100]. This last finding contrasts with the results of the present study, clearly pointing out that MIH is a risk factor for caries in both the deciduous and permanent dentitions [100].

The first permanent molars that have MIH are subject to the possible evolution of lesions over time. As already extensively documented in the literature, colorimetric alterations in MIH have prognostic value, as yellow-brown demarcated opacities have a faster evolution toward enamel breakdown and dentin exposure [9,101].

Children with MIH should be monitored at shorter intervals and should be followed up with a preventive therapeutic approach that can treat hypersensitivity in order to allow proper dental hygiene of mineralized surfaces and soft tissues and with a focus on household lifestyles in order to reduce risk factors for dental caries. The use of organic-based toothpastes with mineralizing and desensitizing agents should be encouraged, as they are claimed to be more biocompatible for children [102].

Preventive measures for dental caries should include: (a) early diagnosis of caries and individual caries risk assessment; (b) methods that focus on enamel and dentin remineralization; (c) minimally invasive treatments; and (d) repair rather than replacement of restoration [103]. In particular, a recent clinical study on the longevity of non-invasive glass ionomer cement, non-invasive and traditional composite restorations, and ceramic restorations on permanent teeth with enamel breakdowns due to MIH showed, over an average period of 4 years, moderate-to-high survival rates for conventional restorations and lower survival rates for non-invasive glass ionomer and composite resin restorations [104].

A decrease in the incidence of dental caries has been demonstrated when prevention is targeted at developmental age in subjects with active but non-cavitated lesions. Applying minimally invasive methodology to routine dentistry leads to an early assessment of caries, which is important in avoiding overtreatment [103].

The process of demineralization and remineralization of enamel constantly moves between mineral loss and gain. The long-term outcome is determined by the composition and amount of dental plaque, frequency and timing of sugar consumption, fluoride exposure, quality and quantity of salivary flow, enamel quality, and individual immune response [8,103].

Repeated consumption of carbohydrates, especially sucrose, leads to the predominance of cariogenic bacteria such as *S. mutans*. These changes in oral biofilm increase the potential for enamel mineral loss, subsequent acid production, and a change in the oral microbiome that leads to an increased risk of developing carious lesions.

The use of sugar substitutes is one measure that helps people reduce their total intake of cariogenic sugars [103].

Furthermore, regenerative medicine deals with dental caries. Several new protocols have been proposed to reconstruct the enamel surface. In situ regeneration of apatite crystals can create thick layers between the enamel and the restoration, while the enamel-like structure due to amelogenins can improve the properties of the etched enamel and prevent dental caries by preventing bacterial infection [105]. For example, carious soft dentin reveals elevated hydrolase, transaminase, and dehydrogenase activity that could originate from invading microbes, saliva, or be endogenous. Pulpal stem cells show alkaline phosphatase activity in areas surrounded by inflamed pulp [106].

Finally, therapeutic strategies should include secondary preventive interventions to be implemented depending on the prognostic factors associated with individual MIH enamel changes and defects to prevent dentin exposure.

Indeed, the authors often point out that since severe forms of MIH are not included in screenings, the DMFT index is often recorded with higher values due to the presence of caries after dentin exposure following post-eruptive enamel breakdown [99].

## 5. Conclusions

The results of the present study, based on a sample size of 17,717 individuals with a mean age of 8.6 years, showed that molar-incisor hypomineralization is significantly correlated with a higher risk of caries in both the permanent and deciduous dentitions in children.

## Data Availability

The data presented in this study are available as supplementary materials or on request from the corresponding author.

## Supplementary Materials

The following supporting information can be downloaded at: https://www.mdpi.com/article/10.3390/healthcare11121795/s1.
